# Mechanistic study of ARHGAP27 promoting the progression of aortic dissection by regulating the RhoA/ROCK/YAP pathway

**DOI:** 10.3389/fcvm.2026.1831795

**Published:** 2026-07-10

**Authors:** Zijie Wang, Jing Tao, Yining Yang

**Affiliations:** 1 College of Life Science and Technology, Xinjiang University, Urumqi, China; 2 Xinjiang Key Laboratory of Cardiovascular Homeostasis and Regenerative Medicine Research, People's Hospital of Xinjiang Uygur Autonomous Region, Urumqi, China; 3 Cardiac and Panvascular Medicine, People’s Hospital of Xinjiang Uygur Autonomous Region, Urumqi, China

**Keywords:** aortic dissection, ARHGAP27, phenotype switch, RhoA/ROCK/YAP signaling, VSMC

## Abstract

**Background:**

Aortic dissection (AD) is a life-threatening cardiovascular disease. ARHGAP27 can regulate cytoskeleton and cellular functions, which may be related to the phenotypic switching of vascular smooth muscle cells (VSMCs) in AD. However, the role of ARHGAP27 in AD has not been reported yet.

**Methods:**

The AD-related data sets were downloaded from GEO database to screen differential genes. HE and IHC staining was used to detect the pathological changes and the expression level of ARHGAP27 in AD tissue. AD cell models were constructed *in vitro* by inducing HAVSMCs with PDGF-BB to evaluate the effects of ARHGAP27 overexpression or knockdown on cell survival, migration, invasion and phenotypic switching. Rescue experiments were performed using the ROCK activator LPA to test pathway specificity.

**Results:**

ARHGAP27 was significantly upregulated in the dataset and the tissue. *In vitro* experiments have shown that overexpression of ARHGAP27 can further promote the survival, migration and invasion of VSMCs, and the expression of synthetic phenotypic proteins MMP2 and MMP9, and inhibit the expression of contraction phenotypes α-SMA and SM22α. Knockdown of ARHGAP27-2 can obtain the opposite result. In terms of mechanism, PDGF-BB down-regulates the expression levels of RhoA, ROCK1/2, and up-regulates the phosphorylation levels of YAP in VSMCs. Overexpression of ARHGAP27 further enhances the effect of PDGF-BB, while knockdown of ARHGAP27-1 and ARHGAP27-2 inhibits the effect of PDGF-BB. Treatment with LPA would reverse the effects produced by ARHGAP27 overexpression.

**Conclusion:**

ARHGAP27 is upregulated in AD and accelerates its pathological progression by regulating the RhoA/ROCK/YAP pathway.

## 1 Introduction

Aortic dissection (AD) is an acute and potentially life-threatening condition characterized by a tear in the intimal layer of the aorta, allowing blood to enter the medial layer and create a false lumen, which may lead to aortic rupture (1). However, its clinical presentation symptoms may not be obvious or overlap with other conditions and not be specific, thus increasing the difficulty of diagnosis (2). As a result, AD is frequently underdiagnosed (3). Because symptoms are usually atypical, mild, or even absent, the clinical suspicion level for AD is often low. Although CT angiography can reliably detect AD, it is frequently postponed when symptoms are unclear, delaying timely imaging (4, 5). Meanwhile, although current European guidelines recommend preventive surgery when the aortic diameter reaches 5.5 centimeters, many AD cases still occur within smaller diameters (6). These limitations highlight the significance of conducting further in-depth research on the potential pathophysiological mechanisms of AD, with the aim of improving the risk prediction models for AD and enhancing the strategies for early diagnosis and treatment.

ARHGAP27 is a member of the Rho GTPase-activating protein (ARHGAPs) family and functions as a negative regulator of Rho GTPases by accelerating the hydrolysis of bound guanosine triphosphate (GTP) to guanosine diphosphate (GDP). Through this regulatory role, ARHGAP27 participates in key cellular processes such as cytoskeletal remodeling, cell migration, contraction, and differentiation (7). Differential expression of ARHGAP27 has been reported in specific tissues and diseases, including spleen, intervertebral disc, chronic lymphocytic leukemia, autism spectrum disorder, pancreatic cancer, and lung cancer, etc (8–11). Given the important role of ARHGAP27 in processes such as cytoskeletal remodeling and cell migration, it is likely involved in the dysfunction of VSMCs and aortic wall remodeling during the pathological process of AD. However, no study has yet investigated the potential role of ARHGAP27 in AD.

Vascular smooth muscle cells (VSMCs) constitute the primary cellular component of the aortic media and are essential for maintaining vascular wall integrity and function (12). The phenotypic switching of VSMCs from the contractile phenotype (differentiated state) to the synthetic phenotype (dedifferentiated state) has been recognized as a key factor in the progression of AD. This process is typically characterized by changes in cell morphology, enhanced cell survival, migration, and invasion capabilities, as well as alterations in the secretion levels of related protein markers (13, 14).

The RhoA/ROCK/YAP pathway is critical in cardiovascular diseases (15, 16). RhoA (Ras homolog family member A) cycles between active GTP-bound and inactive GDP-bound states; its effectors ROCK (Rho-associated coiled-coil containing protein kinase, include two isoforms: ROCK1 and ROCK2) regulate cytoskeletal dynamics and YAP (Yes-associated protein) phosphorylation, influencing cell survival and migration (17, 18). And previous studies reported downregulation of the RhoA/ROCK1/YAP axis in AD (19).

This study aims to validate the differential expression of ARHGAP27—identified through bioinformatics analysis—as a candidate gene associated with AD, at both clinical and *in vitro* VSMC levels. Furthermore, we also attempted to initially explore the molecular pathways by which ARHGAP27 influences the progression of AD, which would help us gain a deeper understanding of its pathogenesis.

## 2 Materials and methods

### 2.1 Materials and reagents

#### 2.1.1 Tissue collection

Normal tissue samples (*n* = 3) were obtained from healthy donors who had died accidentally without heart and aortic diseases, while AD tissue samples (*n* = 5) were collected during the surgical procedures of AD patients. None of the patients had received preoperative adjuvant therapy. This study was approved by the Ethics Committee of Xinjiang Uygur Autonomous Region People's Hospital (Approval No. KY2023042008). All participants provided written informed consent.

#### 2.1.2 Cells

Human aortic vascular smooth muscle cells (HAVSMCs) were purchased from the American Type Culture Collection (ATCC, catalog number: ATCC-CRL-1999).

#### 2.1.3 Reagents

Primary antibodies, ARHGAP27, α-SMA, SM22α, MMP2, MMP9, RhoA, ROCK1, ROCK2, YAP, p-YAP and GAPDH monoclonal antibodies, as well as second antibody, HRP-conjugated goat anti-rabbit IgG, Alexa Fluor 488- and Alexa Fluor 594-conjugated goat anti-rabbit IgG, were obtained from Abcam (Cambridge, UK). Batch numbers: ab171973, ab7817, ab10135, ab92536, ab76003, ab187027, ab134181, ab125025, ab205270, ab76252, and ab181602, as well as ab6721, ab150113, and ab150084, respectively. HRP-labeled goat anti-rabbit IgG antibody was purchased from Lianke Biotechnology Co., Ltd. (Hangzhou, China), batch number: GAR007. ARHGAP27 interference and overexpression plasmids were obtained from Ruibo Biotechnology Co., Ltd. (Guangzhou, China). Cell transfection primer sequence was listed in Table 1. Transwell chambers were purchased from Corning (Costar), batch number: 3422. The CCK-8 kit was obtained from Dojindo Laboratories (Kumamoto, Japan), batch number: CK04. RIPA lysis buffer, BCA protein assay kit, apoptosis detection kit, hematoxylin-eosin staining (H&E) kit, and Active Rho Pull-down and Detection Kit were purchased from Beyotime Biotechnology (Shanghai, China), with batch numbers P0013B, P009, C0003, C0105S, and P2065S, respectively. Lysophosphatidic acid (LPA) was purchased from Avanti Polar Lipids Inc. (Alabaster, USA), with batch number A85328. Platelet-derived growth factor-BB (PDGF-BB) was purchased from MedChem Express (Monmouth Junction, USA), with batch number HY-P73351.

**Table 1:** Primer sequences.

primer	The sequence is 5′→3′
oe-ARHGAP27 Forward	GGACTCAGATCTCGAGGCCACCATGCAGCCGGGCCTGAGC
oe-ARHGAP27 Reverse	TAGAGTCGCGGGATCCTCAGTGCGGCGGGAAGATG
sh-ARHGAP27-1 Forward	GATCCCGGGAAGCCATACTTCTACAACTCGAG TTGTAGAAGTATGGCTTCCCG TTTTTT
sh-ARHGAP27-1 Reverse	CTAGAAAAAACGGGAAGCCATACTTCTACAACTCGAG TTGTAGAAGTATGGCTTCCCG G
sh-ARHGAP27-2 Forward	GATCCCCATCCAGAAGCTACGCTATACTCGAG TATAGCGTAGCTTCTGGATGG TTTTTT
sh-ARHGAP27-1 Reverse	CTAGAAAAAACCATCCAGAAGCTACGCTATACTCGAG TATAGCGTAGCTTCTGGATGG G

### 2.2 Methods

Bioinformatics analysis of GEO datasets (GSE147026, GSE235995, GSE183997) identified differentially expressed genes, which were validated using GSE190635. HE staining and Immunohistochemistry (IHC) staining were performed on human aortic tissues (normal, *n* = 3; AD, *n* = 5) to assess morphological changes and ARHGAP27 expression.

HAVSMCs were stimulated with 20 ng/mL PDGF-BB to establish an AD cell model. ARHGAP27 overexpression and knockdown were achieved by plasmid transfection. The survival, migration and invasion abilities of the cells were detected by CCK-8, scratch and Transwell assays to explore the effect of ARHGAP27 on the function of VSMCs. Expression of contractile markers (α-SMA, SM22α), synthetic markers (MMP2, MMP9), and RhoA/ROCK/YAP pathway proteins (ARHGAP27, RhoA, ROCK1, ROCK2, YAP, p-YAP) was analyzed by Western blot. Additionally, a rescue experiment using the ROCK activator LPA (10 μmol/L) was performed to confirm pathway specificity (see Supplementary Results).

Detailed experimental procedures are provided in the Supplementary Material.

### 2.3 Statistical analysis

All experimental data were analyzed using GraphPad Prism version 8.0. Data were expressed as mean ± standard deviation. Comparisons between two groups (e.g., Control vs. PDGF-BB, sh-NC vs. Sh-ARHGAP27-2) were performed using Student's *t*-test, while one-way ANOVA followed by Tukey's *post hoc* test was used for comparisons among multiple groups (e.g., Control, PDGF-BB, PDGF-BB + oe-ARHGAP27). The “Control” group refers to HAVSMCs cultured without PDGF-BB or transfection. A *p-value* < 0.05 was considered statistically significant.

## 3 Results

### 3.1 ARHGAP27 was significantly upregulated in AD chips and tissue samples

**Figure 1:**
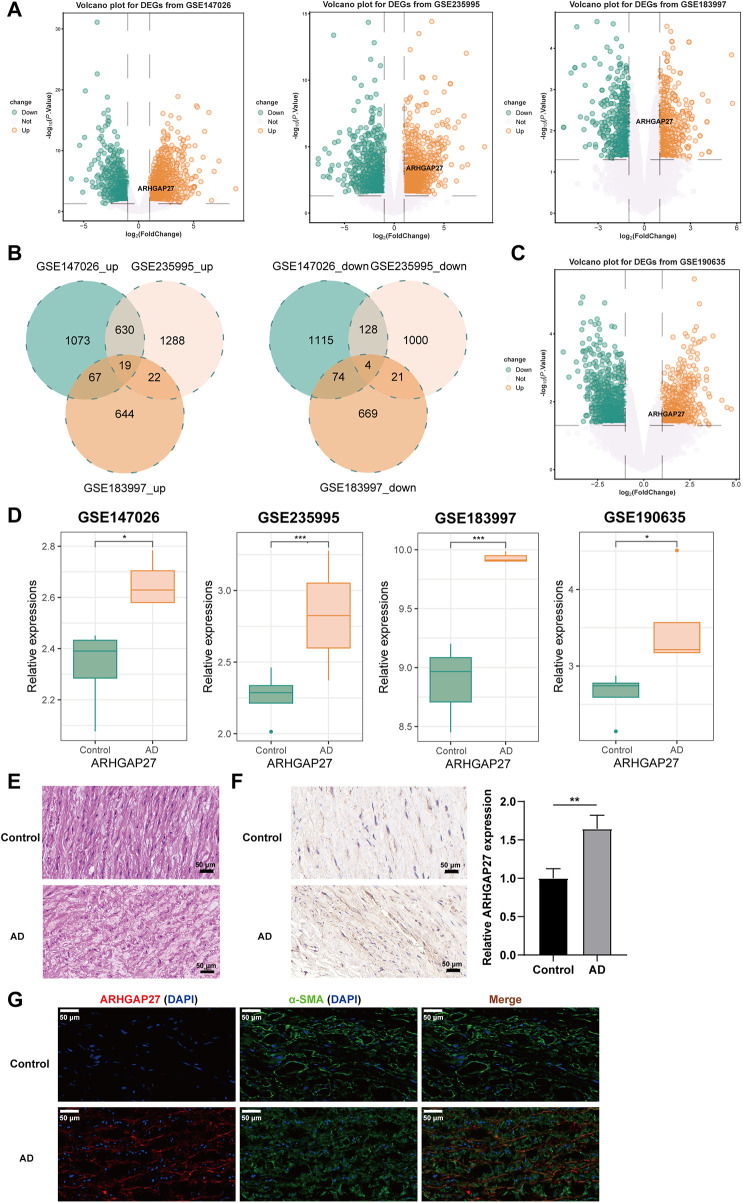
ARHGAP27 was significantly upregulated in AD chips and tissues samples. **(A)** Volcano plot of differential genes screened based on GSE147026, GSE235995, and GSE183997 datasets. **(B)** Venn diagram of differential genes. **(C)** Validation volcano plot using GSE190635 dataset. **(D)** Expression of ARHGAP27 in GSE147026, GSE235995, GSE183997, and GSE190635 datasets. **(E)** HE staining of normal aortic tissue samples and AD tissue samples. **(F)** IHC staining was used to detect the expression of ARHGAP27 in tissues. **(G)** Immunofluorescence co-staining of ARHGAP27 and α-SMA in human aortic tissues. Representative confocal images showing ARHGAP27 (red), α-SMA (green), and nuclei stained with DAPI (blue) in normal and AD aortic tissues. Scale ba*r* = 50 μm. * indicates *P* < 0.05, ** indicates *P* < 0.01, *** indicates *P* < 0.001.

Based on the GEO datasets GSE147026, GSE235995, and GSE183997 (using thresholds of |logFC| > 1, *P* < 0.05), a total of 3,110, 3,112, and 735 differentially expressed genes were identified, respectively (Figure 1A).

The intersection of these gene sets was then analyzed. A total of 19 significantly upregulated co-expressed genes (ITGAX, PLAUR, ARHGAP22, COTL1, PIK3AP1, PODNL1, CTSL, UHRF1, PNP, HMGA1, CLEC5A, MIF, THBS2, TGM2, ARHGAP27, IFITM10, NRIP3, SLC29A3, CLDN7) and four significantly downregulated genes (ACADL, TIMP3, PDE4D, FAM107A) (Figure 1B). Further validated using the GSE190635 dataset, which revealed 1,176 differentially regulated genes (Figure 1C). Notably, ARHGAP27 was consistently upregulated across all four datasets—GSE147026, GSE235995, GSE183997, and GSE190635—with statistically significant *P*-values (Figure 1D, all *P* < 0.05). Therefore, ARHGAP27 is hypothesized to potentially function as a key molecular player in the progression of AD.

Based on the results of bioinformatics analysis, we conducted histological evaluations on both normal aortic tissue samples and AD tissue samples obtained clinically. The HE staining results revealed that normal aortic tissues exhibited an intact and continuous lumen, well-organized elastic fibers, and VSMCs with regular morphology and orderly arrangement. In contrast, the AD group displayed structural disruptions, including cavities within the elastic fibers and disorganized cellular architecture (Figure 1E). Subsequently, immunohistochemical (IHC) staining was performed to assess the expression levels of ARHGAP27 in the tissue samples. The results demonstrated a significantly higher expression of ARHGAP27 in AD tissues compared to normal aortic tissues (Figure 1F, *P* < 0.01). To further determine the cellular localization of ARHGAP27 in aortic tissues, immunofluorescence double staining was performed using antibodies against ARHGAP27 and the VSMCs marker α-SMA. In normal aortic tissues, ARHGAP27 expression was weak and showed limited overlap with α-SMA-positive cells. In contrast, AD tissues exhibited markedly increased ARHGAP27 fluorescence signals that predominantly co-localized with α-SMA-positive cells, indicating that ARHGAP27 is highly expressed in VSMCs during AD progression (Figure 1G).

### 3.2 The effect of ARHGAP27 overexpression on the function of VSMCs and the rhoA/ROCK/YAP pathway

**Figure 2:**
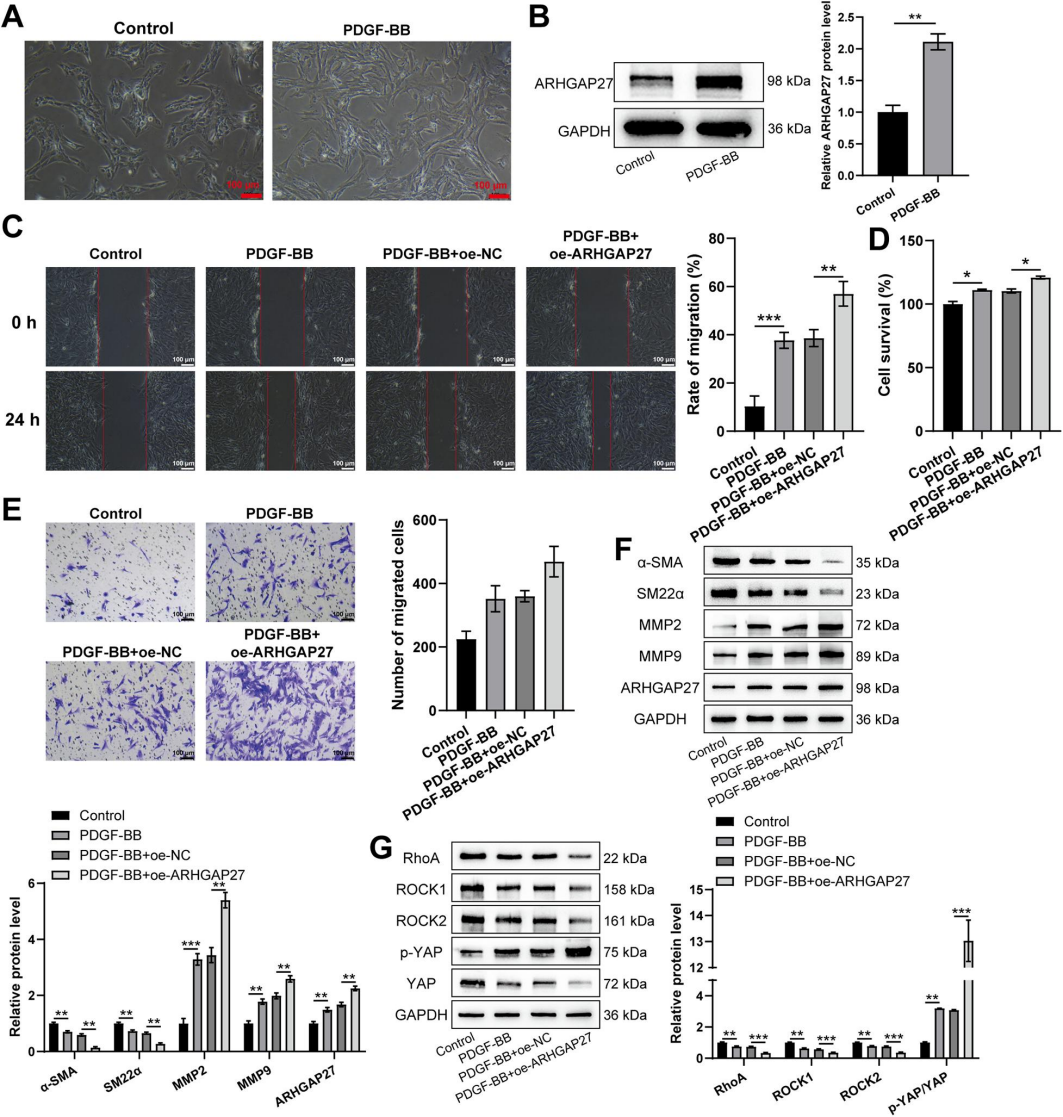
The effect of ARHGAP27 overexpression on the function of VSMCs and the RhoA/ROCK/YAP pathway. **(A)** Observe the cell morphology under microscope. **(B)** Western blot was used to detect the expression of ARHGAP27 in the cells. **(C)** The scratch assay was used to detect the cell migration ability. **(D)** CCK-8 was used to detect cell survival. **(E)** Transwell assay of cell invasion ability (100 μm). **(F)** Western blot analysis of α-SMA, SM22α, MMP2, MMP9 and ARHGAP27 expression in cells. **(G)** Western blot was used to detect the effect of overexpression of ARHGAP27 on the expression of RhoA, ROCK1, ROCK2 and the phosphorylation level of YAP. * indicates *P* < 0.05, ** indicates *P* < 0.01, *** indicates *P* < 0.001.

The effect of ARHGAP27 on AD was further verified through *in vitro* cell experiments. Firstly, HAVSMCs cells were stimulated with PDGF-BB to construct the AD cell model, and the cell morphology was observed under a microscope. The control group had a normal morphology and was spindle-shaped, while the cells in the model group gradually became polygonal, indicating the successful construction of the model (Figure 2A). Furthermore, PDGF-BB induction significantly upregulated the expression level of ARHGAP27 in the cells (Figure 2B, *P* < 0.01). Subsequently, ARHGAP27 overexpression was achieved via plasmid transfection. The results of the CCK-8 assay, scratch assay and Transwell assay indicated that compared with the Control group, cell survival, migration and invasion increased after induction by PDGF-BB, while overexpression of ARHGAP27 could further promote cell survival, migration and invasion (Figures 2C–E, all *P* < 0.05). The results of Western blot indicated that, compared with the Control group, PDGF-BB would reduce the expression of proteins α-SMA and SM22α related to the contractile phenotype in cells and upregulate the expression of proteins MMP2 and MMP9 related to the synthetic phenotype. Overexpression of ARHGAP27 would further promote the effect of PDGF-BB (Figure 2F, all *P* < 0.01).

To investigate whether ARHGAP27 affect AD via the RhoA/ROCK/YAP signaling pathway, this study examined the expression levels of key proteins in this pathway following overexpression of ARHGAP27. The results demonstrated that, compared with the control group, PDGF-BB treatment significantly downregulated the expression of RhoA, ROCK1, and ROCK2, while increasing the phosphorylation level of YAP. Overexpression of ARHGAP27 markedly enhanced these effects induced by PDGF-BB (Figure 2G, all *P* < 0.01).

To further explore the causal relationship between ARHGAP27 and the RhoA/ROCK/YAP pathway, we treated ARHGAP27-overexpressing VSMCs with the ROCK activator LPA. LPA treatment significantly reversed the enhanced survival, migration, and invasion abilities of VSMCs caused by ARHGAP27 overexpression (Supplementary Figures S1A–C), while restoring the expression of contraction phenotype markers α-SMA and SM22α, and inhibiting the expression of synthetic phenotype markers MMP2 and MMP9 (Supplementary Figure S1D). The pathway detection results showed that LPA effectively restored the protein expression levels of ROCK1/2, and reduced the p-YAP/YAP ratio (Supplementary Figure S1E). The GTP pull-down results showed that ARHGAP27 overexpression significantly reduced RhoA-GTP levels, and LPA treatment reversed this effect (Supplementary Figures S1F). This indicates that when the RhoA/ROCK/YAP pathway is artificially activated, the pro-AD effects produced by ARHGAP27 overexpression are significantly weakened. This further proves that the pro-AD effect of ARHGAP27 depends on the inhibition of this pathway.

### 3.3 The effect of ARHGAP27 knockdown on the functions of VSMCs and the RhoA/ROCK/YAP pathway

**Figure 3:**
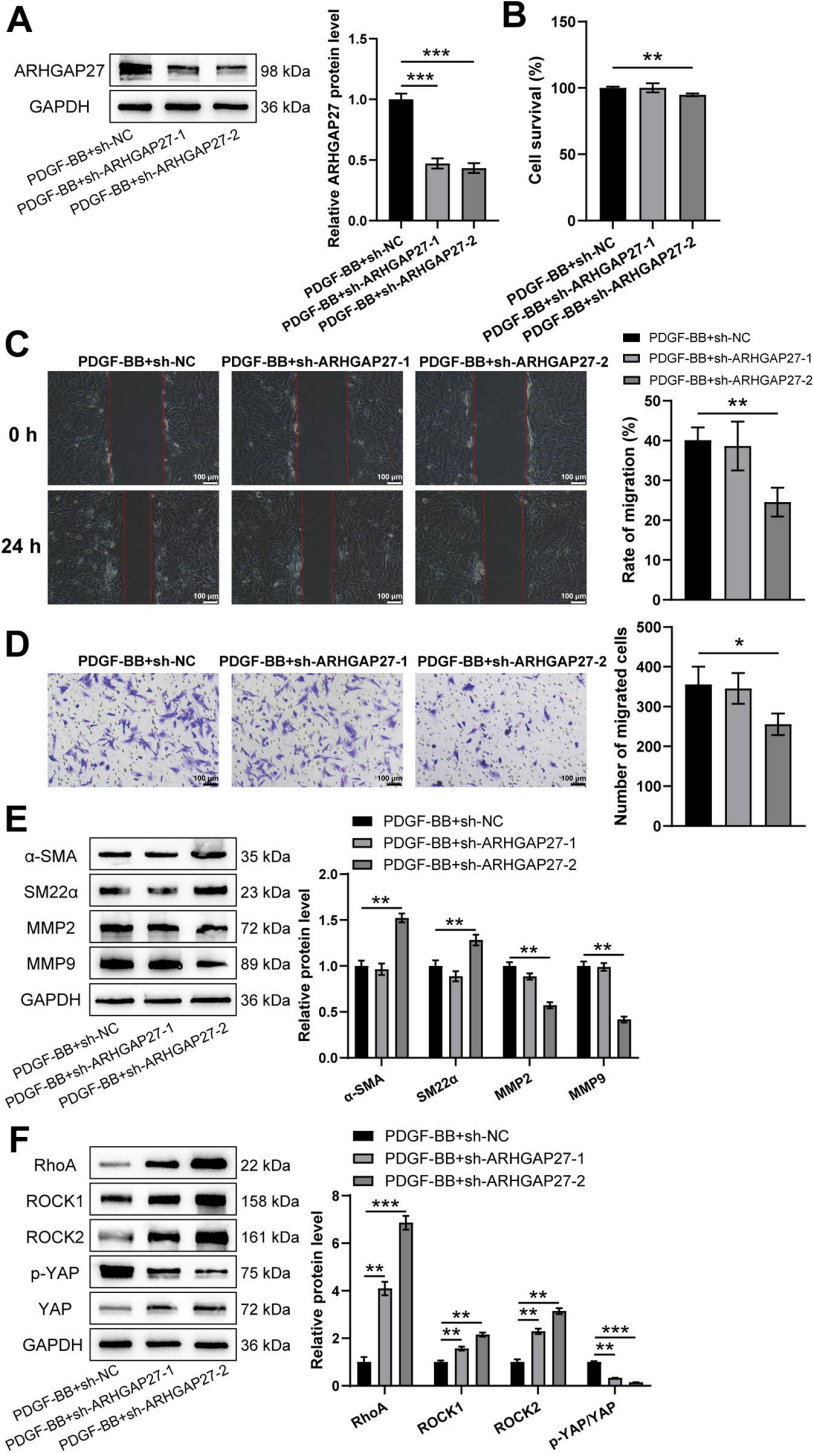
The effect of ARHGAP27 knockdown on the functions of VSMCs and the RhoA/ROCK/YAP pathway. **(A)** Western blot was used to detect the expression of ARHGAP27 in the cells. **(B)** CCK-8 was used to detect cell survival. **(C)** The scratch assay was used to detect the cell migration ability. **(D)** Transwell assay for cell invasion ability. **(E)** Western blot analysis of α-SMA, SM22α, MMP2 and MMP9 expression. **(F)** Western blot was used to detect the effect of knocking down ARHGAP27 on the expression of RhoA, ROCK1, ROCK2 and the phosphorylation level of YAP. * indicates *P* < 0.05, ** indicates *P* < 0.01, *** indicates *P* < 0.001.

ARHGAP27 expression was suppressed using cell transfection techniques. Due to alternative splicing of the promoter region, ARHGAP27 has two isoforms: ARHGAP27-1 and ARHGAP27-2 (20). Therefore, knockdown experiments were conducted separately on ARHGAP27-1 and ARHGAP27-2. The transfection efficiency is presented in Figure 3A. Compared with ARHGAP27-1, the knockdown effect of ARHGAP27-2 was more effective (Figure 3A). Results from the CCK-8 assay, scratch assay, and Transwell assay demonstrated that, in comparison to the sh-NC group, silencing ARHGAP27-2 significantly inhibited cell survival, migration, and invasion (Figures 3B–D, all *P* < 0.05), whereas knockdown of ARHGAP27-1 did not yield statistically significant differences (Figures 3B–D, all *P* > 0.05). Western blot analysis revealed that, compared to the sh-NC group, suppression of ARHGAP27-2 led to increased expression of contractile phenotype-related proteins α-SMA and SM22α, while decreasing the expression of synthetic phenotype-related proteins MMP2 and MMP9 (Figure 3E, both *P* < 0.01). In contrast, no significant changes were observed following knockdown of ARHGAP27-1 (Figure 3E, *P* > 0.05). Silencing either ARHGAP27-1 or ARHGAP27-2 attenuated the regulatory effects of PDGF-BB on the expression of RhoA, ROCK1, ROCK2, and on YAP phosphorylation (Figure 3F, all *P* < 0.05).

## 4 Discussion

In this study, ARHGAP27 was identified as a significantly upregulated gene in AD across four GEO datasets and validated in human AD tissues by IHC staining. Functional experiments demonstrated that overexpression of ARHGAP27 promotes the survival, migration, invasion of VSMCs, and the phenotypic switching from the contractile to the synthetic phenotype, while knockdown results in the opposite effect. Mechanistically, ARHGAP27 negatively regulates the RhoA/ROCK/YAP axis, which is supported by the decreased expression of RhoA, ROCK1/2 and increased phosphorylation of YAP when ARHGAP27 is overexpressed. Importantly, the rescue experiments using the ROCK activator LPA reversed all the phenotypic and molecular changes caused by ARHGAP27 overexpression. This provides reverse evidence that ARHGAP27 affects the function and phenotypic switching of VSMCs in AD by negatively regulating RhoA/ROCK/YAP.

ARHGAP27 belongs to the RhoGAP family, and several members of this family have been shown to be related to vascular diseases. For example, ARHGAP18 can prevent atherosclerosis by regulating the alignment of vascular endothelial cells (EC) in the direction of flow, and maintain vascular homeostasis through Rho-mediated microtubule regulation of the endothelial cell barrier function (21, 22). ARHGAP24 can regulate the proliferation and dedifferentiation of VSMCs, and is related to neointimal hyperplasia diseases such as restenosis and atherosclerosis (23). However, the role of ARHGAP27 in AD has not been reported yet. Our findings extend the current understanding of RhoGAP proteins to the pathogenesis of AD, providing a new potential direction to address the current issue of inadequate diagnostic approaches for AD. Additionally, recent discoveries have also indicated that the RhoA/ROCK/YAP pathway is inhibited in AD (19). Our research further discovered that ARHGAP27 might be a new and important upstream negative regulator of this pathway in AD.

The significant upregulation of ARHGAP27 in AD suggests that it may serve as a new biomarker or therapeutic target. However, this study still has some limitations. Firstly, the clinical sample size was small (*n* = 5 AD, *n* = 3 normal), but the expression difference of ARHGAP27 reached statistical significance and was consistent with four independent GEO datasets. Secondly, our AD cell model (VSMCs stimulated by PDGF-BB) cannot fully simulate the complex mechanical forces of the aortic wall. Thirdly, we did not conduct animal studies; the causal relationship and *in vivo* correlation need to be verified by a vascular smooth muscle cell-specific ARHGAP27 knockout mouse model. These limitations are common in exploratory studies. Future research will expand the sample cohort and conduct more in-depth validation in AD mouse models by specifically deleting ARHGAP27 in VSMCs. At the same time, conducting chromatin immunoprecipitation sequencing (ChIP-seq) or other more in-depth mechanism studies will help us gain a deeper understanding of the specific molecular mechanisms by which ARHGAP27 regulates the RhoA/ROCK/YAP pathway. Studying the impact of ARHGAP27 on the microfibrillar tissue in AD and its potential role in extracellular matrix remodeling will also be very meaningful. Additionally, single-cell RNA sequencing of human AD tissues may reveal the cell source of ARHGAP27, and the mechanical stretching system may better simulate the AD microenvironment.

In summary, this study first demonstrated that ARHGAP27 is upregulated in AD and affects the function and phenotypic switching of VSMCs by negatively regulating the RhoA/ROCK/YAP pathway. The rescue experiments provided more powerful evidence for this regulatory relationship. Although further *in vivo* validation is needed in the future, our current findings provide a new approach for exploring the pathogenesis of AD and potential therapeutic targets.

## 5 Conclusion

In conclusion, ARHGAP27 is upregulated in AD and correlates with enhanced VSMCs survival, migration, invasion, and phenotypic switching in PDGF-BB-induced cellular models. The signaling pathway experiments and rescue experiments indicate that ARHGAP27 negatively regulates the RhoA/ROCK/YAP axis. While this study provides preliminary clinical and *in vitro* insights, further *in vivo* investigations are required to establish causality and clinical relevance.

## Data Availability

The original contributions presented in the study are included in the article/Supplementary Material, further inquiries can be directed to the corresponding author.
