# Supplementary material for: Mechanistic study of ARHGAP27 promoting the progression of aortic dissection by regulating the RhoA/ROCK/YAP pathway
Source: Front Cardiovasc Med. 2026 Jul 10;13:1831795. doi: 10.3389/fcvm.2026.1831795 (PMC13396006; doi:10.3389/fcvm.2026.1831795)
Supplement: Supplementary file 1 [file Table1.docx]

**Supplementary Materials**

**1. Supplementary Method**

**1.1 Bioinformatics Analysis of Differentially Expressed Genes**

The GSE147026 dataset (GPL24676 platform, 4 controls and 4 AD tissues), GSE235995 (GPL24676 platform, selecting 2 controls and 4 AD tissues), and GSE183997 (GPL21827 platform, 3 controls and 3 AD tissues) were downloaded from the Gene Expression Omnibus (GEO) database (http://www.ncbi.nlm.nih.gov/geo/). Differentially expressed genes were identified by intersecting these three datasets and further validated using the GSE190635 dataset (GPL570 platform, 4 controls and 4 AD tissues).

**1.2 Hematoxylin and Eosin (H&E) Staining**

The tissue samples were fixed in 4% paraformaldehyde, dehydrated using a graded ethanol series, and cleared with xylene. Paraffin-embedded sections (4 μm thickness) were prepared. After dewaxing and rehydration with graded ethanol, the sections were stained with hematoxylin and eosin for 10 minutes, dehydrated again, cleared with xylene, mounted with neutral resin, and examined under a light microscope.

**1.3 Immunohistochemistry (IHC) Staining**

The paraffin sections were dewaxed, rehydrated, and subjected to antigen retrieval using EDTA solution. Sections were blocked with 3% bovine serum albumin (BSA) for 30 minutes. Anti-ARHGAP27 primary antibody was applied and incubated overnight at 4 °C. After washing with PBS, HRP-conjugated goat anti-rabbit IgG secondary antibody (1:200) was added, and sections were incubated at room temperature for 1 g. Following PBS washing, sections were incubated with fresh prepared DAB solution for 15 min and further counterstained with hematoxylin. Sections were mounted with mounting medium and observed under an optical microscope.

**1.4 Immunofluorescence staining**

To determine the cellular localization of ARHGAP27 in human aortic tissues, immunofluorescence double staining was performed on paraffin-embedded aortic sections obtained from normal controls and AD patients. After deparaffinization, rehydration, and antigen retrieval in citrate buffer, the sections were blocked with 5% goat serum and incubated overnight at 4°C with primary antibodies against ARHGAP27 (1:100) and α-smooth muscle actin (α-SMA, 1:200), a specific marker of VSMCs. After washing with PBS, the sections were incubated with the corresponding Alexa Fluor 488- and Alexa Fluor 594-conjugated secondary antibodies for 1 h at room temperature. Nuclei were counterstained with DAPI, and fluorescence images were captured using a confocal fluorescence microscope. Co-localization of ARHGAP27 and α-SMA signals was analyzed to evaluate ARHGAP27 expression in medial VSMCs.

**1.5 Cell culture and AD model construction**

HAVSMCs were cultured in SmGM-2 medium with 5% fetal bovine serum (FBS) at 37 °C, 5% CO₂. When cell confluence reached approximately 80%, cells were subjected to serum starvation by culturing in medium containing 0.5% FBS for 24 hours. Subsequently, an AD cell model was established by stimulating the cells with 20 ng/mL platelet-derived growth factor-BB (PDGF-BB), and morphological changes were observed under a microscope.

**1.6 Cell transfection**

Cells were seeded into 24-well plates at a density of 2 × 10⁵ cells/mL. sh-ARHGAP27-1, sh-ARHGAP27-2, and oe-ARHGAP27 plasmids were transfected into the cells to achieve ARHGAP27 knockdown and overexpression, respectively. All transfection procedures were performed in accordance with the manufacturer's instructions for Lipofectamine 3000.

**1.7 Cell Function Assays**

**Cell Counting Kit-8 (CCK-8):** Cells (5 × 10⁴ cells/mL, 100 μL/well) were seeded in 96-well plates. CCK-8 reagent (10 μL/well) was added and incubated for 2 h. Absorbance at 450 nm was measured.

**Scratch test to detect cell migration:** Cells grown to 70-80% confluence in 6-well plates were starved (serum-free, 24 h). A scratch was made with a 200 μL pipette tip, and migration was observed microscopically.

**Transwell Invasion Assay:** Cells (2 × 10⁵ cells/mL, 200 μL) in serum-free DMEM were added to the upper chamber (pre-coated with Matrigel) of the Transwell insert; the lower chamber contained 10% FBS. After 24 h, invading cells were fixed, stained with crystal violet, and counted.

**1.8 Western blot detection of protein expression**

Tissue homogenates and cells were lysed in RIPA buffer with protease inhibitors, followed by protein concentration determination with the BCA Protein Quantification Kit. The proteins were separated via 10% SDS-PAGE and transferred to PVDF membranes. The membranes were blocked at room temperature for 2 hours with 5% BSA to prevent non-specific binding. Primary antibodies (rabbit monoclonal) ARHGAP27, α-SMA, SM22α, MMP2, MMP9, RhoA, ROCK1, ROCK2, YAP and p-YAP were added at appropriate concentrations, with GAPDH as the internal control. After overnight incubation at 4 ℃, the membrane was washed and treated with HRP-labeled goat anti-rabbit secondary antibody IgG for 1 h at room temperature. ECL working solution was used for detection. Western blot images were quantified using Image Pro Plus 6.0 software for gray-scale analysis of each groups bands, with all experiments repeated three times.

**1.9 Rescue experimental groups and treatments**

HAVSMCs were seeded in 6-well plates (cell density 2 × 10^5^ cells per well), and when the cell confluence reached 70%-80%, they were treated according to the following groups:

(1) Control group: Normal culture without any treatment;

(2) PDGF-BB group: Added 20 ng/mL PDGF-BB for 24 hours;

(3) PDGF-BB + oe-NC group: Transfected with overexpressed empty plasmid (oe-NC) for 24 hours, then added 20 ng/mL PDGF-BB for 24 hours;

(4) PDGF-BB + oe-ARHGAP27 group: Transfected with oe-ARHGAP27 plasmid for 24 hours, then added 20 ng/mL PDGF-BB for 24 hours;

(5) PDGF-BB + oe-ARHGAP27 + LPA group: Transfected with oe-ARHGAP27 plasmid for 24 hours, then added 20 ng/mL PDGF-BB and 10 μmol/L LPA (lysophosphatidic acid, a ROCK agonist) for co-treatment for 24 hours.

After the treatments of each group were completed, CCK-8 assay, scratch assay, Transwell assay and Western blot detection were conducted. The indicators for Western blot detection included: VSMC phenotypic markers (α-SMA, SM22α, MMP2, MMP9) and pathway-related proteins (RhoA-GTP, ROCK1, ROCK2, p-YAP/YAP).

**1.10 GTP pull-down assay to detect RhoA-GTP**

The level of active RhoA (RhoA-GTP) was determined using an Active Rho Pull-down and Detection Kit according to the manufacturer’s instructions. Briefly, cells were washed twice with ice-cold PBS and lysed in the supplied lysis buffer containing protease inhibitors on ice. After centrifugation at 12,000 × g for 10 min at 4°C, the supernatants were collected and protein concentrations were determined using a BCA assay. Equal amounts of protein lysates were incubated with Rhotekin-RBD agarose beads at 4°C for 1 h with gentle rotation to selectively precipitate GTP-bound RhoA. The beads were then washed three times with wash buffer, resuspended in SDS loading buffer, and boiled for 5 min. The precipitated proteins and total cell lysates were subsequently subjected to Western blot analysis using an anti-RhoA antibody. The level of active RhoA was quantified as the ratio of RhoA-GTP to total RhoA.

**2. Supplementary Figures**

**Supplementary Figure S1. The effect of adding ROCK activator LPA on the functional abnormalities and phenotypic transformation of VSMCs overexpressing ARHGAP27 in vitro cell models. (A)** CCK-8 was used to detect cell survival. **(B)** The scratch assay was used to detect the cell migration ability. **(C)** Transwell assay for cell invasion ability. **(D)** Western blot analysis of α-SMA, SM22α, MMP2 and MMP9 expression. **(E)** Western blot was used to detect the expression levels of ROCK1, ROCK2 and the phosphorylation level of YAP. **(F)** GTP pull-down assay was used to detect the activity of RhoA. Note: * indicates *P* < 0.05, ** indicates *P* < 0.01, *** indicates *P* < 0.001.
