# Supplementary figures and images for: Mechanistic study of ARHGAP27 promoting the progression of aortic dissection by regulating the RhoA/ROCK/YAP pathway
Source: Front Cardiovasc Med. 2026 Jul 10;13:1831795. doi: 10.3389/fcvm.2026.1831795 (PMC13396006; doi:10.3389/fcvm.2026.1831795)

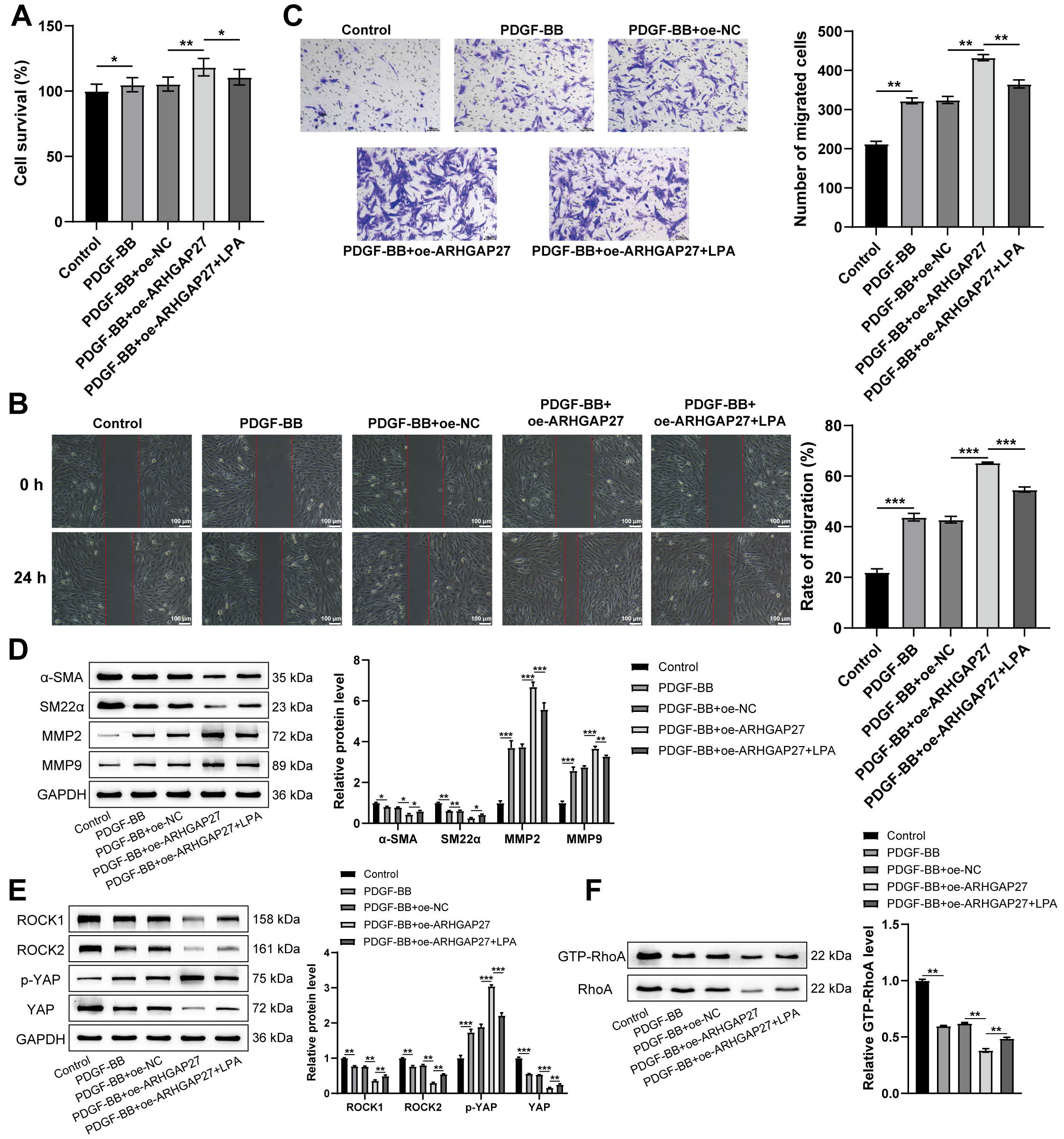

Supplement: Supplementary file 2 [file Image1.tif]
